# Su(z)2 Antagonizes Auto-Repression of Myc in Drosophila, Increasing Myc Levels and Subsequent Trans-Activation

**DOI:** 10.1371/journal.pone.0005076

**Published:** 2009-03-31

**Authors:** Abid Khan, Wesley Shover, Julie M. Goodliffe

**Affiliations:** Department of Biology, The University of North Carolina, Charlotte, North Carolina, United States of America; Institute of Genetics and Molecular and Cellular Biology, France

## Abstract

All tumor cell lines that have been tested are defective for Myc auto-repression, and have high levels of Myc produced from wild type loci and re-arranged loci. Like mammalian Myc auto-repression, Myc protein represses the expression of its gene, dmyc, in Drosophila. This activity requires Polycomb (Pc), since RNAi for Pc in the embryo eliminates Myc auto-repression. We have observed that upon depletion of Polycomb in the embryo, levels of one of 18 different chromatin-binding genetic regulators, Su(z)2, rise dramatically. We pursued the possibility that increased levels of this protein, Su(z)2, interfere with Myc auto-repression, potentially explaining the loss of auto-repression upon Pc RNAi. We report that embryos expressing both ectopic Myc and ectopic Su(z)2 fail in Myc auto-repression. Surprisingly, histone H3K27 tri-methylation at the dmyc locus is inversely correlated with the presence of auto-repression. We show phenotypic consequences of potent dmyc auto-repression, and their complete reversal by ectopic Su(z)2: dmyc auto-repression induced a diminutive (dm) phenotype, and upon elimination of auto-repression by Su(z)2, overall levels of Myc increased and completely rescued the phenotype. We show that this increase in Myc levels caused dramatic activation of Myc activation targets. These data suggest that Su(z)2 is capable of increasing the potency of Myc activity by eliminating Myc's feedback regulation by auto-repression. Although Su(z)2 eliminated Myc auto-repression, we found that Myc repression of other genes is not affected by Su(z)2. These data suggest a unique antagonistic role for Su(z)2 in Myc auto-repression, and a potential mechanism for cancer-cell specific loss of Myc auto-repression.

## Introduction

Myc protein is required for cell growth and proliferation in Drosophila and mammals, and its function is conserved among all metazoans, with the exception of nematodes [1], [2], [3]. Myc affects transcription by inducing or repressing hundreds, if not thousands, of target genes; Myc protein can bind to 11% of all human promoters, with some sites bound in almost all cells and some sites bound only at high Myc levels [4]. Genes regulated by Myc include those that promote growth and proliferation and inhibit differentiation [5]. Independent of DNA binding, Myc can affect the phosphorylation state of the C-terminal domain of RNA polymerase II, affecting RNA processing and translation [6]. Over-expression of Myc also affects DNA replication, modulating origin of replication activity and leading to replication stress and DNA damage [7]. These activities of Myc explain its potent oncogenic activity.

Although equally important, the mechanism of Myc's activity of transcriptional repression is less well understood than its mechanism of transcriptional activation. Myc is capable of repressing targets when recruited to a promoter via another transcription factor, such as Miz-1 [8]. In addition, Myc and its heterodimerization partner, Max, have been found to bind to promoters repressed by Myc independently of Miz-1 [9], [10], [11]. A truncated version of Myc that fails in transcriptional activation is still functional in repression, suggesting a mechanism of trans-repression that is distinct from activation and does not require Myc's activation of co-repressors [12]. Newly discovered targets of Myc repression include a set of microRNA genes, the repression of which contributes to tumor formation. The promoters of these genes are bound by Myc protein [13]. Therefore, binding of Myc to promoters is important for repression, however the mechanism by which it is recruited there is unknown.

Myc is capable of repressing its own gene, and this auto-repression is disrupted in all cancer cell lines tested [14], [15]. We have shown previously that the chromatin binding repressor Polycomb (Pc) is required for Myc to repress its own gene, dmyc, in Drosophila [16]. Polycomb Group (PcG) proteins are known for their role in regulation of gene expression necessary for cellular differentiation, stem cell maintenance, and avoidance of tumorigenesis [17], [18]. Polycomb group proteins work in large, multi-subunit complexes that bind to and modify chromatin, silencing hundreds of loci from flies to mammals [18], [19], [20], [21], [22], [23], [24], [25]. Pc RNAi in the Drosophila embryo results in the loss of the majority of trans-repression by Myc, among many other gene expression changes [16], [26]. Along with the decrease in repression by Myc, we report here an increase in levels of Su(z)2, a PcG related protein [27], [28], [29], [30] that is required for ectopic Myc-induced overgrowth in the Drosophila eye [31]. We show that an increase in Su(z)2 alone disrupts auto-repression by Myc, but not Myc repression of other targets. As a consequence of the loss of auto-repression, we show that elevated levels of ectopic Myc lead to an increase in Myc *activation* of its targets. These data provide the first evidence for a protein that interferes with Myc auto-repression, leading to elevated Myc levels and subsequent increases in activation by Myc.

## Results

### Su(z)2 up-regulation results in the loss of auto-repression by Myc

**Figure 1:**
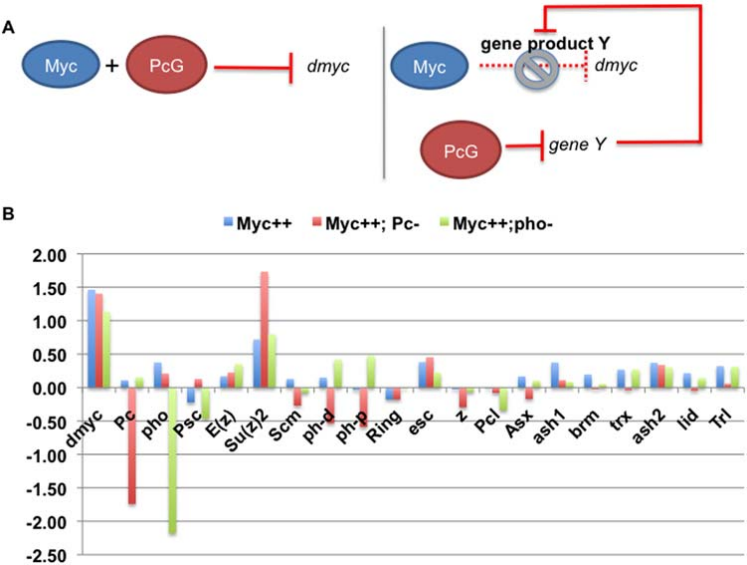
Pc RNAi results in an increase in Su(z)2 levels. A) We suggest two possible scenarios for the role of Pc in Myc auto-repression: Pc is either directly involved with Myc protein in repression (left), or required to repress a third player, gene Y (right), whose protein product interferes with Myc repression (right). B) A candidate for gene Y. See Goodliffe et al., 2007, for microarray data generation. Log_2_ ratios of changes in levels of 19 PcG and TrxG transcripts are shown, compared to wild type levels (Gal4), in embryos with ectopic Myc (blue), ectopic Myc plus Pc RNAi (red) and ectopic Myc plus pho RNAi (green).

Ectopic Myc expression in Drosophila embryos results in the repression of 80–200 genes, including the Drosophila myc (dmyc) gene. Depletion of Polycomb by RNAi abrogates auto-repression and the majority of Myc repression at mid embryogenesis [16], [26]. A logical hypothesis for the role of Polycomb in Myc repression is that it directly binds to and represses these targets. However, this hypothesis is inconsistent with our previous results indicating that a PcG protein required for the targeting of Polycomb, Pho, does not play the same role in repression by Myc. Many Myc repression targets affected by Polycomb are unaffected by depletion of Pho [26]. Because Pho physically targets Polycomb to many loci [32], [33], [34], we considered the possibility that Polycomb is not directly targeted to all genes that Myc represses. Therefore, we hypothesized that the role of Polycomb in repression by Myc is indirect: Polycomb is required to repress a different gene or genes, and that repression is required for Myc-induced repression (Figure 1A).

If the role of Polycomb in repression by Myc is largely indirect, then our genome-wide expression data [16], [26] should provide information regarding the gene or genes in the Myc-Polycomb repression pathway. We began a search for candidate genes by examining gene expression changes in any genes known to be generally involved in chromatin binding and genetic regulation. Therefore, we examined gene expression changes of many PcG and Trithorax Group (TrxG) genes upon Pc RNAi in the embryo. Among 18 such genes (pho, Psc, E(z), Su(z)2, Scm, ph-d, ph-p, Sce/Ring, esc, z, Pcl, Asx, ash1, brm, trx, ash2, lid and Trl), only Su(z)2 levels changed significantly upon Pc RNAi: Su(z)2 transcripts *increased* 4-fold upon Pc RNAi (Figure 1B, microarray data are described in Goodliffe et al. 2007). These data indicated that Pc is required for repression of Su(z)2, which is consistent with previous reports of cross-regulation among PcG members [35]. We were intrigued by these data, because Su(z)2 is a functional homolog of the PcG protein Psc, and known to be a potent repressor when targeted to loci by LexA fusion [29], [36], [37], [38]. Our data suggested the potential for Su(z)2 to affect auto-repression by Myc, and despite the unexpected potential for a repressor to interfere with repression, we pursued the possibility.

We considered two hypotheses: 1) that Pc is required for direct repression by Myc and Su(z)2 levels are incidental, or 2) that Su(z)2 blocks repression by Myc and Polycomb is required to repress the Su(z)2 gene (Figure 1A, where gene Y represents Su(z)2). To determine whether the latter hypothesis is true, we obtained flies from the Exelixis collection that contain an XP insertion near the endogenous Su(z)2 locus (P[XP]d01221, abbreviated here as Su(z)2XP) [39], leading to its ectopic expression under the control of Gal4 as detected by RT-PCR (data not shown). We obtained embryos expressing ectopic dmyc, ectopic Su(z)2, and the combination of both under the control of an armadillo-Gal4 driver (see Materials and Methods for genetic crosses).

**Figure 2:**
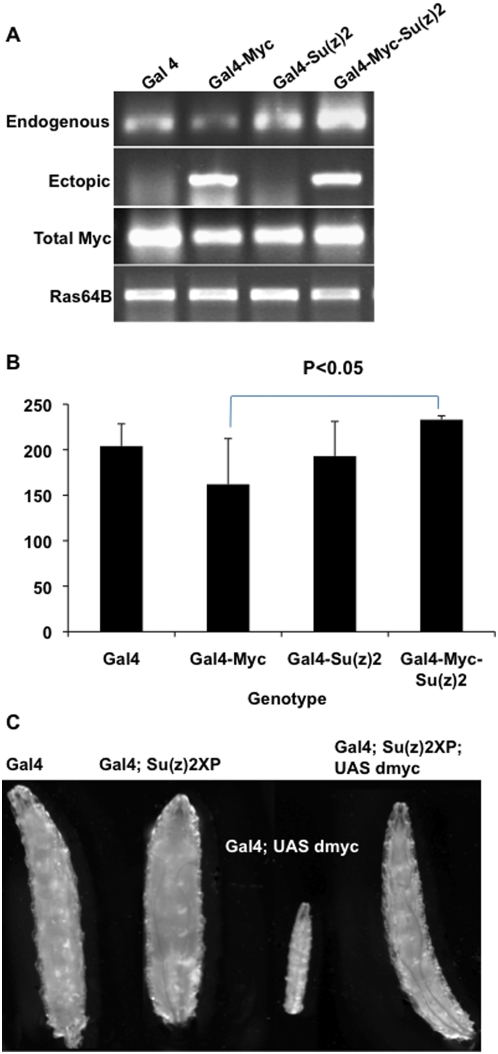
Su(z)2 disrupts auto-repression by Myc. A) RT-PCR analysis of endogenous, ectopic and total Myc expression in embryos of four different genotypes, which are indicated above each lane (Gal4 = arm-Gal4, Gal4-Myc = armGal4; UAS dmyc, Gal4-Su(z)2 = armGal4; Su(z)2XP, Gal4-Myc-Su(z)2 = armGal4; Su(z)2XP; UAS dmyc). A 0–21 hour collection of embryos was used for RNA isolation and for all subsequent assays. Ras64B was used as a loading control. B) A chart showing endogenous dmyc expression, the average of biological triplicates is plotted with standard deviations indicated for four genotypes of embryos. Expression was quantified using Quantity 1 (Bio-Rad). The blue line denotes a statistically significant change in endogenous dmyc levels from Gal4-Myc to Gal4-Myc-Su(z)2. C) Living larvae of the genotypes shown, all grown at low density, aged 4 days after egg laying at room temperature, and photographed simultaneously.

In embryos with ectopic Myc, endogenous dmyc gene expression was reduced compared to wild type. In contrast, simultaneous ectopic Myc and ectopic Su(z)2 expression resulted in a dramatic *increase* in endogenous dmyc expression compared to levels in embryos expressing ectopic Myc alone (P = 0.036 by Student's t-test, Figure 2A–B). These results suggest that, at the minimum, Su(z)2 expression interferes with dmyc auto-repression. Because endogenous dmyc levels were also increased over wild type levels, ectopic Su(z)2 and Myc together appear to cause an activation of endogenous Myc in addition to relief of auto-repression. In fact, increased levels of Su(z)2 alone appear to induce endogenous dmyc, though to a lesser degree than with ectopic Su(z)2 and Myc together. These results suggest the importance of wild type dmyc auto-repression, which when disrupted, may allow induction of the dmyc gene in the embryo potentially by the mitogenic signals that are likely to be operating during embryogenesis.

**Figure 3:**
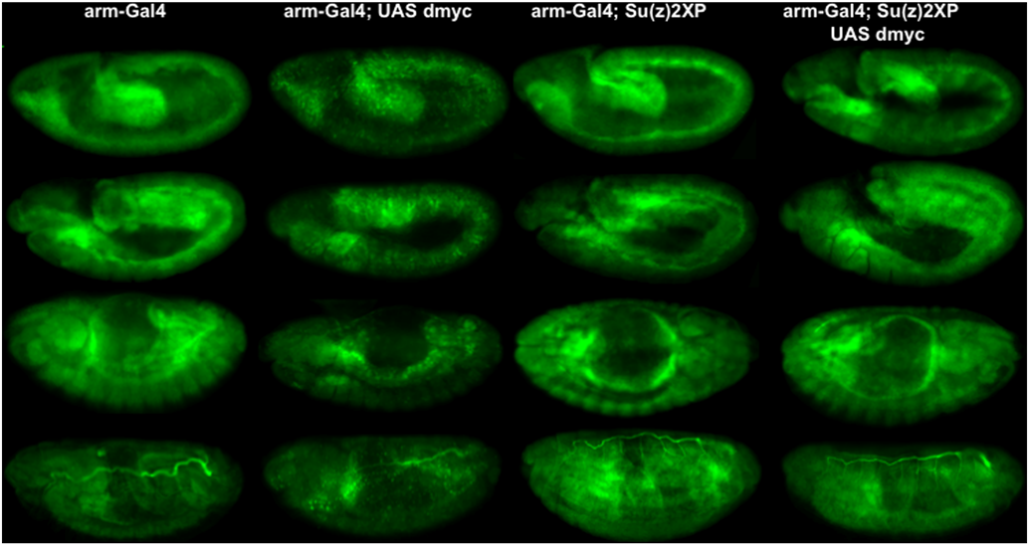
dmyc auto-repression reduces total Myc protein levels compared to wild type, which is rescued by combining ectopic Myc with ectopic Su(z)2 expression. Similarly aged embryos of four genotypes are beside one another, with the genotypes of embryos indicated above each column. Anti-Myc staining is green.

We considered the possibility that Su(z)2 may interfere with Myc protein levels, accounting for the failure of auto-repression. We obtained an antibody specific for Drosophila Myc, and compared its staining with fluorescent in situ hybridization (FISH) to dmyc transcripts in wild type embryos (data not shown and Fly-FISH [40]). Because we observed the same staining pattern with both FISH and immuno-staining using the anti-Myc antibody, we examined levels of Myc protein in embryos of four genotypes (armGal4, armGal4-UAS dmyc, armGal4-Su(z)2XP, armGal4-UAS dmyc-Su(z)2XP). We found that ectopic Su(z)2 does not decrease Myc protein levels (Figure 3). In fact, we found that embryos expressing ectopic Myc have reduced total Myc protein levels, and those expressing both ectopic Myc and ectopic Su(z)2 have wild type levels of Myc (Figure 3). These results are consistent with our RT-PCR data and indicate that increased levels of Su(z)2 provide increased Myc protein levels in the absence of auto-repression.

In these experiments, we found that armGal4 provided low levels of ectopic Myc compared to wild type levels found in embryos. As shown in Figures 2–3, total Myc levels are high in Gal4, non-ectopic Myc expressing embryos. In embryos with ectopic Myc, endogenous Myc levels dropped dramatically, and therefore the total Myc levels must have consisted of mostly ectopic Myc. When the levels of ectopic Myc were lower than Myc levels that occur in wild type embryos, transgenic, dmyc auto-repressing embryos had lower total Myc than wild type embryos (Figures 2 and 3). These data suggest that auto-repression can reduce the naturally occurring, high levels of Myc to the point that what remains in those embryos is ectopic Myc. If ectopic Myc is low, total levels are low in those embryos. Consistent with these low levels of Myc protein, we observed a phenotypic consequence of the growth of these animals.

In support of our molecular data, we observed that larvae heterozygous for Gal4 and UAS dmyc resembled larvae zygotically null for dmyc [41]. In these experiments, we used a Gal4 driver that allowed more embryos to survive and hatch into larvae (P{Gal4}-da.G32, [42]) than survive with armGal4-UAS dmyc. Many Gal4-da.G32-UAS dmyc embryos hatched into viable larvae, however these larvae failed to grow, and after approximately 3 days, died as small larvae (Figure 2C). These larvae behaved as wandering third instars, and often died attached to the side of the vial. Larvae heterozygous for Gal4, UAS dmyc and UAS Su(z)2 together, however, hatched and grew normally, completely rescued presumably by the inhibition of auto-repression by Su(z)2 (Figure 2C). These data illustrate the impact of strong dmyc auto-repression, which is to induce a dmyc knock-down phenotype, and that Su(z)2 appears to completely rescue this mutant phenotype.

### General repression by Myc is maintained and possibly enhanced by Su(z)2

We were curious whether ectopic Su(z)2 interferes with general repression by Myc at genes other than dmyc. We tested the expression of 6 known embryonic repression targets of Myc (Cyp6a8, CG31274, Obp56a, CG12868, CG31445, JhI-26 [16], [26]) by RT-PCR using total RNA from embryos of the four genotypes described above (Gal4, Gal4-UAS dmyc, Gal4-Su(z)2XP, Gal4-UAS dmyc-Su(z)2XP). Two interesting results were evident from these experiments.

**Figure 4:**
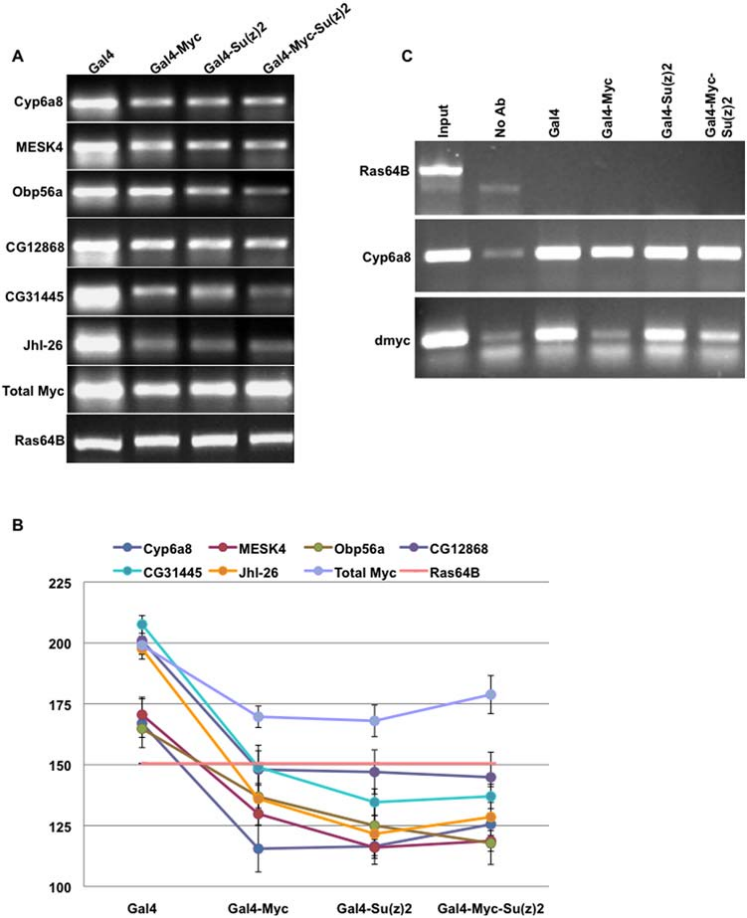
Ectopic Su(z)2 does not interfere with Myc repression of targets other than dmyc. A) A representative gel of RT-PCR data showing the expression of six Myc targets of repression in four genotypes, as indicated above each column of bands. Total dmyc expression and Ras64B expression, a loading and RNA level control, are shown in the bottom two panels. B) The average band intensities indicating levels of expression and relative standard deviation are plotted for the 8 genes shown in A. Genotypes are indicated along the X axis, and the Y axis shows band intensities as quantified by Quantity 1 (Bio-Rad). C) ChIP results showing H3K27 tri-methylation of three of the 8 genes shown in A, in the 4 genotypes of the experiment. Ras64B is a negative control. These data show a representative picture of biological triplicates for all 8 genes.

First, all six genes were repressed in embryos with ectopic Myc, despite a slight drop in overall Myc levels caused by auto-repression (Figure 4A–B). This result occurred repeatedly and for all 6 genes we tested. These data suggest that this set of six genes is repressed by Myc in a mechanism that occurs before or simultaneously with Myc repression of its own gene.

Second, these genes were also repressed in embryos with ectopic Su(z)2, either alone or in combination of ectopic Su(z)2 and ectopic Myc (Figure 4A–B). These results were also observed repeatedly for six of six tested genes, and suggest that Su(z)2 not only fails to interfere with repression by Myc but also appears to replace it. One possible explanation for repression of these genes in the presence of ectopic Su(z)2 alone is the status of endogenous dmyc auto-repression in these embryos. Total Myc levels are elevated in embryos with ectopic Su(z)2 alone compared to Gal4-UAS dmyc embryos (Figures 2–3), suggesting the disruption of endogenous dmyc auto-repression. It is possible that the disruption of auto-repression causes increased Myc levels at a time when these genes are sensitive to repression by Myc. Alternatively, Su(z)2 alone may be sufficient for repression of these targets. Taken together, our results show that Su(z)2 interferes with auto-repression by Myc, but Su(z)2 appears not to affect and possibly to enhance repression of other Myc targets.

Gene activation or repression can be a consequence of or maintained by specific covalent histone modifications [43]. Tri-methylated lysine 27 on histone 3 (H3K27-Me3) is a well defined mark of PcG repression [44] and found to be present at Myc activation and repression targets in wild type embryos [16], [26]. To test whether Su(z)2 affects this chromatin modification at Myc repression targets, we performed chromatin immunoprecipitation (ChIP) using antibodies specific for H3K27-Me3 (Millipore) in four genotypes. We were surprised to find no difference in methylation levels of the 6 repression targets in any of the four genotypes. All six were consistently tri-methylated at H3K27 in all four genotypes. We were further surprised to find reduced H3K27-3Me at the dmyc locus in embryos with ectopic Myc expression, inversely correlated with auto-repression (Figure 4C). These data suggest that embryonic H3K27 tri-methylation at these loci does not mediate their expression or repression. However, the reduction of H3K27 tri-methylation of dmyc in embryos expressing both ectopic Myc and ectopic Su(z)2 is intriguing. One possibility to explain these results is that Myc auto-repression utilizes a mechanism that involves removal of PcG complexes from the locus.

### Activation by Myc is enhanced by Su(z)2

**Figure 5:**
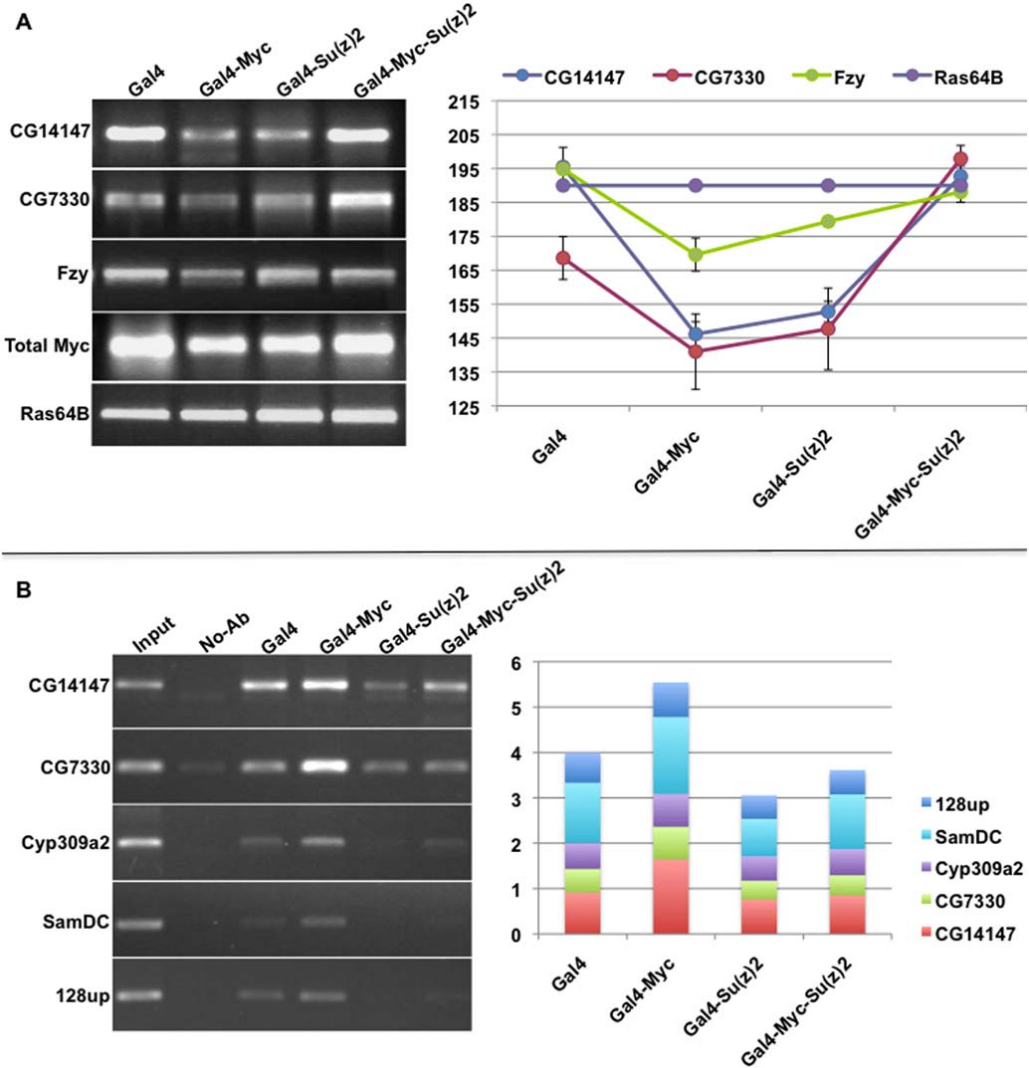
Increased Su(z)2 provides for activation of Myc targets, and a reduction in their H3K27 tri-methylation. A) RT-PCR analysis showing expression of three Myc activation targets (indicated on the left side of the gel pictures) in embryos of 4 different genotypes (indicated above the lanes). Average band intensities (Quantity 1) of biological triplicates are plotted on the right, with relative standard deviations indicated. B) ChIP assay showing histone H3K27 tri-methylation at five Myc activation targets (indicated to the left of the gel pictures) in embryos of genotypes indicated on top. Data shown on the left are plotted in a stacked column chart; the y-axis is the density of each PCR product divided by the density of the input sample PCR product, and the values for each gene are stacked together for each of the four genotypes. These data show a representative set of biological triplicates.

We were interested in the downstream consequences of the elimination of Myc auto-repression by Su(z)2, especially considering the dramatic phenotypes we observed in larvae. We tested the expression of three known targets of Myc *activation* (CG14147, CG7330 and Fzy [26]) in embryos of the four genotypes utilizing the armGal4-UAS system as described above. Expression of ectopic Myc led to auto-repression and therefore reduced endogenous Myc levels. Upon reduction of endogenous Myc, total Myc levels, consisting of the remaining endogenous plus ectopic Myc, were lower in embryos expressing ectopic Myc than wild type (Figure 5A). As a result, levels of all three tested downstream activation targets were reduced in the effectively Myc-knock-down embryos (Figure 5A). In embryos expressing both ectopic Myc and Su(z)2, total Myc levels were high, similar to levels in wild type embryos. Accordingly, the levels of all three Myc activation targets are elevated, similar to their levels in wild type embryos. We observed these results repeatedly, and using embryos of various ages. These data show that, in embryos with both ectopic Myc and ectopic Su(z)2, the loss of auto-repression leads to an overall increase in Myc levels, which is sufficient to activate downstream Myc targets. These gene expression changes help explain the phenotypic rescue of Gal4-UAS dmyc-Su(z)2XP animals.

We have previously published reports of strong activation by Myc of hundreds of genes in embryos [16], [26]. The difference between those experiments and the ones we report here is that levels of ectopic Myc are much lower in our newest experiments than they have been previously. Because levels of ectopic Myc are low, and because we still obtain auto-repression of the endogenous locus, we have lower overall levels of Myc in embryos with ectopic Myc compared to wild type, as explained above. This results in a decrease in Myc activation of Myc targets. Therefore, our data show the consequences of de facto knock-down of Myc levels on Myc targets, which is that they are de-activated. The results are consistent with these targets being Myc responsive, since their levels drop as Myc levels drop. Our data are in agreement with previous results, and add the novel result that Su(z)2 disruption of auto-repression can alter downstream Myc function.

### Ectopic Su(z)2 alters histone H3 lysine 27 methylation at Myc targets

We have shown previously that embryonic targets of Myc *activation* are tri-methylated at H3K27 [26]. To test whether Su(z)2 affects H3K27 chromatin modification at Myc activation targets, we performed chromatin immunoprecipitation (ChIP) using antibodies specific for H3K27-Me3 (Millipore). We used embryos aged 0–21 hours, maintaining identical conditions to our RT-PCR experiments shown in Figure 5A, and used ChIP DNA as template for PCR amplification of several genes: CG14147, CG7330, Cyp309a2, SamDC and 128up. The first two represent the set that were activated with ectopic Myc and Su(z)2 (Figure 5A). Cyp309a2 is activated by Myc at mid embryogenesis but not later [Goodliffe, unpublished and 16,26], and SamDC and 128up have both been reported to respond to Myc in Drosophila and have Myc bound at their promoters [45].

In the absence of ectopic Myc, four out of five Myc activation targets were methylated at H3K27 (Figure 5B). Upon ectopic Myc induction, and under conditions of de-activation caused by a reduction in total Myc levels (as in Figure 5A), all five loci showed increased H3K27 methylation (Figure 5B). These results indicate that reduced Myc levels lead to increased H3K27-3Me at Myc activation targets. Consistent with a Su(z)2-induced increase in total Myc levels, HeK27-Me3 decreased in embryos having both ectopic Myc and Su(z)2 (Figure 5B). These data are consistent with our RT-PCR results and indicate that activation of these targets involves reduced H3K27 tri-methylation, and that ectopic Su(z)2 may impact this reduction. Interestingly, at three of the five loci, ectopic Su(z)2 alone reduces H3K27-Me3 levels compared to their levels in Gal4 embryos (CG14147, Cyp309a2, 128up).

## Discussion

Su(z)2 has two mammalian homologs: Bmi-1 and Mel-18 [29], [38]. Bmi-1 was originally isolated as a collaborator with Myc in tumorigenesis [46], [47], and Myc has been shown to directly activate Bmi-1 [48]. Interestingly, Mel-18 behaves as a tumor suppressor by repressing oncogenes Bmi-1 and c-myc in mammals [49], [50]. However, Mel-18 and Bmi-1 have both been shown to increase proliferation and survival of cancer cells [51].

We have shown that auto-repression of dmyc expression in embryos is a potent mechanism that can persist throughout embryogenesis, and that Su(z)2 disrupts this mechanism. These results explain, in part, the role of Polycomb in Myc auto-repression, which involves suppression of Su(z)2 expression. We had previously reported that Pc RNAi up-regulated many genes that are also up-regulated upon *increased* total Myc levels [16]. Our data suggest that the intermediate between those sets of genes is likely to be Su(z)2: Su(z)2 levels increase without Pc, followed by the disruption of dmyc auto-repression, leading to an increase in Myc levels and the subsequent activation of Myc targets.

We are intrigued that a repressor interferes with repression, and specifically the auto-repression of the dmyc gene and not other Myc repression targets. Our results suggest different mechanisms for dmyc auto-repression and repression by Myc in general. In the former case, the expression of Su(z)2 somehow interferes with auto-repression mediated by ectopic Myc, and in the latter case, Su(z)2 appears not to affect and possibly replace repression by ectopic Myc. Because it is unlikely that a potent repressor such as Su(z)2 directly interferes with repression, we suppose that its role in interference with auto-repression is indirect. There may be a gene that is responsive to the combination of elevated Su(z)2 and elevated Myc, and this gene product may interfere with auto-repression.

We have not ruled out the possibility, however, that the over-expression of Su(z)2 caused it to replace Psc in the Polycomb Repressive Complex 1 (PRC1). Although Psc and Su(z)2 have been shown to be functional homologs in that complex [37], an embryonic Su(z)2-PRC1 complex may be targeted differently than a PRC1 complex containing Psc, and therefore potentially remove other components of the PRC1 complex from the dmyc locus. It is also possible that increased Su(z)2 protein levels titrate members of PRC1, causing non-functional complexes to accumulate and interfere with functional complexes.

We use whole embryos for our experiments because we are interested in the endogenous, embryonic mechanisms that allow an embryo to tolerate high levels of Myc. We have shown that auto-repression is potent and can induce a dmyc knock-down phenotype. We suspect that the wild type role of Su(z)2's interference of dmyc auto-repression is important for the prevention of complete silencing of dmyc. Embryos require Myc protein for growth, and our results show that Su(z)2 helps maintain expression of dmyc, providing Myc levels necessary for embryonic growth and proliferation.

## Materials and Methods

### Drosophila stocks and crosses

We crossed females homozygous for a Gal4 driver (armGal4 [52] or Gal4-da.G32 [42]) to males homozygous for UAS dmyc [53], Su(z)2XP (P[XP]d01221, see flybase.org for further information), and UAS dmyc-Su(z)2XP. As controls, we crossed Gal4 females to Gal4 males.

### Microarray data normalization and analysis

The microarray data plotted in Figure 1 has been published previously; see Goodliffe et al. (2007) for normalization and data treatment.

### RNA isolation and RT-PCR

We conducted crosses as described, and collected embryos on grape juice agar plates (Genesee) supplemented with live yeast. Total RNA was isolated using TRIzol (Invitrogen), and RNA samples were DNAse treated (Promega) prior to RT-PCR. We used AccessQuick RT-PCR system (Promega) to amplify target transcripts from RNA, and quantified band intensities using Quantity 1 (Bio-Rad). PCR cycles were minimized to examine expression changes within the linear range (24–25 cycles, depending on the primer set).

We isolated RNA from embryos aged 0–21 hours, and examined expression levels of endogenous dmyc expression using a primer set that amplifies the 5′ untranslated region of dmyc, which is absent in the UAS dmyc transgene [16]. To amplify ectopic myc expression, we used a primer that binds to the 9E10 epitope tag present on the transgenic transcript. All experiments were done in biological triplicates, with no more than 25 PCR cycles.

### Chromatin immunoprecipitation

ChIP was performed as described previously [16], [26]. We used antibodies at a 1∶100 concentration, amplified target genes using GoTaq Hot-Start polymerase (Promega), and quantified band intensities using Quantity 1 (Bio-Rad). Anti-H3K27-3Me is obtained from Millipore, 07-449.

### Embryo fixation, staining and microscopy

Embryos were fixed in formaldehyde/PBS, and stained using 1∶250 concentration of anti-Myc (Santa Cruz) in PBS/0.1% Triton X-100/5% BSA. Secondary antibodies were used at a 1∶500 dilution (AlexaFluor 488 anti-rabbit, Invitrogen). Embryos were mounted in SlowFade Gold with DAPI (Invitrogen), and imaged using a Motic BA400 compound microscope, Lumen 200 Illumination Systems epi-fluorescence, Spot Cooled CCD monochrome camera and software. We photographed all embryos with identical bulb intensity and acquisition settings.
